# Dual Regulation of Dendritic Morphogenesis in *Drosophila* by the COP9 Signalosome

**DOI:** 10.1371/journal.pone.0007577

**Published:** 2009-10-26

**Authors:** Inna Djagaeva, Sergey Doronkin

**Affiliations:** Department of Anatomy and Neurobiology, University of Tennessee Health Science Center, Memphis, Tennessee, United States of America; Katholieke Universiteit Leuven, Belgium

## Abstract

Altered dendritic arborization contributes to numerous physiological processes including synaptic plasticity, behavior, learning and memory, and is one of the most consistent neuropathologic conditions found in a number of mental retardation disorders, schizophrenia, and neurodegenerative disease. COP9 signalosome (CSN), an evolutionarily conserved regulator of the Cullin-based ubiquitin ligases that act in the proteasome pathway, has been found associated with diverse debilitating syndromes, suggesting that CSN may be involved in regulation of dendritic arborization. However, the mechanism of this control, if it exists, is unknown. To address whether the CSN pathway plays a role in dendrites, we used a simple and genetically tractable model, *Drosophila* larval peripheral nervous system. Our model study identified the COP9 signalosome as the key and multilayer regulator of dendritic arborization. CSN is responsible for shaping the entire dendritic tree through both stimulating and then repressing dendritic branching. We identified that CSN exerts its dualistic function via control of different Cullins. In particular, CSN stimulates dendritic branching through Cullin1, and inhibits it via control of Cullin3 function. We also identified that Cullin1 acts in neurons with the substrate-specific F-box protein Slimb to target the Cubitus interruptus protein for degradation.

## Introduction

Dendritic spine morphogenesis is a fundamental part of the process of synapse formation and maturation during brain development. Dendritic spine dysfunction and degeneration are thought to be among the earliest events in Alzheimer's disease, correlate with cognitive deficits in Alzheimer's disease patients, and occur in animal models [1], [2]. Morphological alterations of dendrites and spines occur in Huntington's disease and in animal models [3], [4], [5]. These data indicate at least an indirect link between spine morphogenesis and disease.

Dendritic pathology also is a consistent feature of mental retardation disorders [6]. At the level of the neuronal network, even modest alterations in dendritic structure and organization of many neurons lead to mental retardation and autism spectrum disorders. In particular, reduction of dendritic complexity, reflecting reduced dendritic branching and the morphology of dendritic spines, was described in Down and Rett syndromes [7]. On the other hand, neuropathological studies have also reported the opposing abnormalities in the mentaly retarded brain. For example, fragile X syndrome is characterized by an increase in dendritic spines with long, thin, immature morphology, suggesting a deficiency in developmental pruning of the spines [6]. Thus, it appears that the “mental disorder neuron” has too many or too few, too strong or too weak, excitatory synapses relative to the level of inhibition [8]. These changes may result in suboptimal neuronal network connectivity and, consequently, intellectual discapacity.

Numerous regulatory pathways have been found to participate in an ever-growing signaling pathway network in dendritic spines [5], [6], [9], [10]. The programmed, rapid, and substrate-specific degradation of proteins by the ubiquitin-proteasome pathway plays a critical role during virtually every event in cellular metabolism, including neurodevelopment [11]. Targeting of proteins for destruction is accomplished by covalent attachment, through an enzymatic cascade, of polymers of the small protein ubiquitin to the protein substrate. These ubiquitin tags serve as a label for proteins that must be degraded after completion of their duties. Polyubiquitin-tagged proteins are then recognized and degraded by the 26S proteasome, a large protease complex [11].

The specificity and timing of ubiquitin attachment to particular protein targets is the responsibility of E3 ubiquitin ligase complexes, acting together with E1 ubiquitin-activating enzymes and E2 ubiquitin-conjugating enzymes [12], [13]. Ubiquitin ligases are particularly important because they recognize proteins that are no longer required and target them for degradation. Two main archetypes of ubiquitin ligases have been identified on the basis of the presence of either HECT domain or a RING-like motif [14].

The architecture of RING (Really Interesting New Gene) ubiquitin ligases is typically based on the assembly of substrate-specific ubiquitin ligase complexes using Cullin family proteins as scaffold. Cullins are highly conserved among species and serve as invariable catalytic core components of E3 enzymes and are associated with a number of variable substrate-specific adaptors that mediate binding and specificity [13]. Among the best-characterized Cullin-RING ubiquitin ligases are Cullin1-based Skip1-Cullin1-F-box (SCF) complexes. Less is known about E3 enzymes based on other Cullin family members. Most higher eucaryotic genomes express five different Cullins – Cul1, Cul2, Cul3, Cul4, and Cul5.

Activity of the Cullin-based E3 complexes is in turn regulated by the protein complex called COP9 (COnstitutive Photomorphogenesis mutant 9) signalosome [15], [16], [17]. The COP9 signalosome (CSN), which is composed of eight subunits (CSN1–8), is an evolutionarily conserved protein complex that has been reported to control pleiotropic functions from yeast to humans [15], [16], [18], [19], [20]. Increasing evidence indicates that CSN-mediated removal of a small ubiquitin-like molecule Nedd8 from Cullin component of ubiquitin ligases (or deneddylation) plays a critical role in regulating Cullin-dependant proteolysis [21], [22], [23]. Cycles of neddylation and deneddylation of Cullins are thought to be required for normal function of Cullin-based ubiquitin ligases [13], [21]. CSN activity has been reported to be critical for the deneddylation of various Cullins in various genetic models [16], [19].

Regulation of a wide variety of events by Cullin-mediated ubiquitination exerts pleiotropic effect of the COP9 signalosome on development [15], [24]. COP9 has been reported to control cell cycle, DNA stability, angiogenesis, and microenvironmental homeostasis that are critical for tumor development [15], [16], [25]. In *Drosophila*, in our and others' experiments, CSN plays a critical role in oogenesis, eye development, in fly immune system and circadian rhythms [18], [20], [26], [27], [28].

Recent active studies in the field of mental disorders and neurodegenerative disease revealed that members of the COP9 signalosome were associated with several neurological disorders. In particular, subunit 3 of the COP9 signalosome, CSN3, was reported to be associated with Smith-Magenis syndrome, a multiple congenital anomaly/mental retardation syndrome in autism spectrum disorders [29], [30], [31], [32]. It appears also that CSN is involved, directly or not, in Down syndrome, one of the most frequently isolated causes of mental retardation. CSN complex subunit 4, CSN4, has been identified aberrantly expressed in Down syndrome brain [33]. A different study has suggested that CSN5, CSN subunit 5, is involved in the onset of neuronal diseases such as Alzheimer's disease and Parkinson's disease [34]. Another CSN subunit, CSN2, has been found to play a critical role in the early stage of neuronal differentiation of embryonal carcinoma cells [35].

Moreover, mutations in the components of the CSN-dependent machinery are also associated with neurological disease. Accumulation of the NEDD8 protein was commonly observed in Lewy bodies in Parkinson's disease and in glial inclusions of Machado-Joseph disease, an autosomal dominant neurodegenerative disease [36], [37]. Alterations in CUL4B, a scaffold protein of E3 ligase, cause an X-linked mental retardation syndrome [38], [39].

Because CSN function appears to be strongly connected to neurodegenerative disease and mental retardation disorders and because of the virtually complete lack of knowledge of the CSN-associated neuropathology, we set out to study a role for CSN in control of dendritic morphogenesis in a simple, tractable and well-tested genetic model system, *Drosophila melanogaster*. *Drosophila* has proved itself to be a powerful model system to study human diseases [40], [41]. To investigate the effect of CSN on dendritic development, we used the larval peripheral nervous system (PNS) that provides an opportunity for easy and convenient monitoring development of the PNS neurons labeled by the green fluorescent protein (GFP) [42]. Using this approach, we found that the CSN machinery is crucial for *Drosophila* dendritic morphogenesis. CSN appears to be in control of the signals that shape the entire dendritic tree.

Here we report that CSN acts to provide the proper balance in dendritic development. Failure in CSN regulation leads to either stimulation or reduction of dendritic branching. Our data demonstrate that CSN exerts its stimulating or inhibiting function via functions of different Cullin-based ubiquitin ligases. In particular, we detected that CSN normally prevents dendritic over-branching via control of the Cullin3-based ubiquitin ligase in degradation of the actin-crosslinking protein Kelch (59). Here we show that CSN acts to stimulate dendritic arborization via the Cullin1-based ubiquitin ligase. Failure in Cullin1 function leads to reduced dendritic branching. We identified the F-box protein Slimb as a substrate-specific component of the Cullin1-based E3 ligase in regulating dendritic morphogenesis. We also found the transcription cofactor protein Cubitus interruptus (Ci) as a downstream target of Cullin1^Slmb^-mediated proteolysis in neurons: Ci is specifically accumulated in *cullin1*-mutant neurons, and overexpression of Ci mimics the *cullin1-* and *slmb-*mutant phenotypes.

## Results

### COP9 signalosome is involved in neuronal development

**Figure 1:**
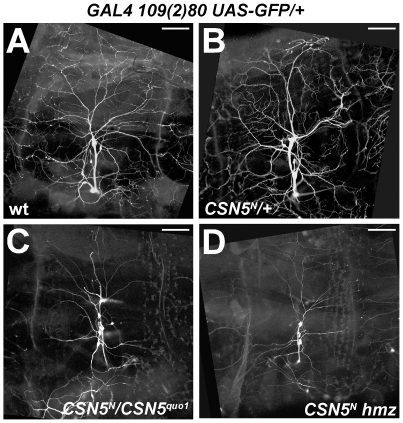
Reduction of *CSN5* alters neuronal development. (A) PNS dendritic arborization neurons in a non-mutant third instar larva, visualized by the *109(2)80-GAL4*-driven expression of UAS-GFP. (B) *CSN5^N^* heterozygotes have normal dendritic arborization. (C) *CSN5^N^*/*CSN5^quo1^* trans-heterozygotes and (D) *CSN5^N^* homozygotes repress dendritic branching. In all figures anterior is oriented to the left. Scale bar: 50 µm.

To investigate whether COP9 signalosome is involved in dendritic development, we used *Drosophila* larval PNS as a model system. Larval PNS neurons elaborate characteristic highly branched sub-epidermal patterns that can be visualized in living embryos or larvae with green fluorescent protein (GFP) [42] (Figure 1A). We visualized dendritic arborization (DA) sensory neurons via *GAL4-109(2)80*-driven expression of *UAS-GFP* in several alleles of the key component of the COP9 signalosome, *CSN5*. *CSN5* is the most evolutionarily conserved subunit of the COP9 signalosome.

*CSN5-*null mutants die at different stages ranging from embryos to pupae [20]. We detected that DA neurons in alive *CSN5*-null homozygous mutant third instar larvae were often less elaborated (Figure 1D), while *CSN5* heterozygotes did not repress dendritic elaboration (Figure 1B). A similar effect was detected in *CSN5^null^/CSN5^quo1^* double heterozygous (Figure 1C). We also found a range in severity of the mutant phenotype: some DA neurons were affected more than others. This range of defects was observed at different stages of larval development. These results indicated that CSN is involved in dendritic development.

#### Mutations in CSN5 lead to the diverse effect on dendritic arborization

We then investigated how CSN is involved in development of the individual neurons. To alter the function of the CSN complex in single neurons, we used the mosaic analysis with repressible cell marker (MARCM) technique [43] that allows generating GFP-labeled mutant clones in otherwise non-mutant larvae.

We found that, at 25°C, loss of *CSN5* in the null *CSN5-*mutant neurons led to severe defects in dendritic branching, making it impossible to identify the type of the mutant neuron based on its morphology (data not shown).

Because we previously found that the effect of *CSN5* mutations is temperature dependent [18], [20], we continued our experiments at a lower, more permissive, temperature. As expected, at 18°C, *CSN5*-null mutant phenotype was less severely defective. At this temperature, general neuronal morphology in *CSN5*-mutants was largely unaffected, and neurons were recognizable. However, this phenotype appeared to be highly complex. We identified two unequal classes in the *CSN5* mutant phenotype.

**Figure 2:**
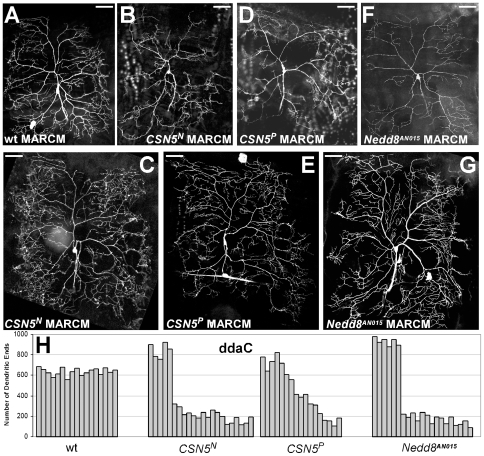
*CSN5* or *Nedd8* mutations have a diverse effect on dendritic branching. (A) Mosaic clone of a wild-type ddaC neuron marked by GFP. (B) In *CSN5^N^* mutants, ddaC neurons frequently show decreased dendritic branching. (C) A fraction of *CSN5^N^* mosaic clones has increased dendritic branching. *CSN5^P^* mutants demonstrate similar repressed (D) or stimulated (E) dendritic arborization phenotype. (F) Mutations in *Nedd8* frequently decrease dendritic elaboration. (G) Loss of *Nedd8* can lead to increased dendritic branching. Scale bar: 50 µm. (H) Quantifications of terminal dendritic ends in wild type, *CSN5^N^*, *CSN5^P^* and *Nedd8* mutant ddaC neurons. Numbers of dendritic ends in individual neurons are shown instead of average value.

The first, larger, class of the *CSN5*-mutant phenotype was characterized by the reduced number of dendritic ends. In particular, normally highly branched ddaC neurons in *CSN5*-mutants developed significantly fewer branches and a shrunken dendritic tree (Figure 2B; 2A – wild type control). Apparently, this phenotype represented a mild version of the severe defects observed at 25°C: more permissive temperature led to the weaker defects in dendritic branching.

Strikingly, lowering the temperature also led to the appearance of another, quite opposite, phenotypic class in *CSN5* mutants: in particular, clones with excessive dendritic branching (Figure 2C). Increased branching caused more dense surface cover and an expanded dendritic field. These changes did not affect the general morphology of the neurons: every neuron was easily recognizable.

These less- and over-branching phenotypes were detected with different frequencies. Mutations in *CSN5* led to more frequent inhibition of dendritic branching and less frequent stimulation of the branching. In *CSN5*-null mutants, we did not detect the “intermediate” phenotype. Remarkably, we were able to detect both phenotypes in the same larva in different neurons, despite the identical genetic background. Furthermore, we observed in the same larva the opposite effect on the same types of neurons.

Parallel MARCM experiments on the hypomorph *CSN5* alleles revealed similar dualistic phenotype. We observed either a reduction or amplification of dendritic branches in ddaC neurons (Figure 2D, E). However, we detected that weaker *CSN5* alleles caused less extreme distribution of the phenotypes. In contrast to *CSN5*-null mutants, loss of *CSN5* in these clones led to a gradient between fewer dendritic ends to their excess: in addition to the severely defective phenotypes in *CSN5* hypomorphic neurons, we also detected an “intermediate” phenotype (Figure 2H).

Taken together, these results suggest that CSN acts cell-autonomously in neurons and regulates morphogenesis in both directions, inhibiting and stimulating dendritic branching. By removing CSN function, we were able to detect both forms of the morphogenesis defects.

#### Loss of nedd8 causes phenotype similar to CSN5

We found that CSN is involved in regulating dendritic morphogenesis. This finding raised a question as to whether the COP9 signalosome is involved in this process by regulating the activity of Cullin-based E3 ubiquitin ligases and, consequently, protein degradation. To address this issue, we used several mutations in components of the CSN-mediated degradation machinery.

CSN controls function of the Cullin-based ubiquitin ligases by removing the Nedd8 modification from Cullins. Cycles of Nedd8 attachments and cleavages are required for the ubiquitin ligase function. To investigate whether the observed dendritic defects were to the result of affected deneddylation in *CSN5* mutants, we generated MARCM clones of strong *Nedd8* mutations.

Detailed analysis of the *Nedd8* phenotype and its comparison to the loss of *CSN5* revealed their principal similarity. We detected that loss of *Nedd8* dramatically suppressed neuronal development at 25°C. In many cases, Nedd8-mutant ddaC neurons demonstrated severe defects in dendritic branching, similar to *CSN5-*mutants. We found two opposite effects of *Nedd8* on dendritic branching at 18°C degrees, also similar to that we found in *CSN5* mutants. We detected the less and more elaborated dendritic phenotypes (Figure 2F, G). These results indicated that CSN and Nedd8 act together in dendritic morphogenesis to regulate proteolysis.

### CSN5 and Nedd8 control DA neurons of different classes

In addition to the ddaC neurons, we found that mutations in *CSN5* or *nedd8* disrupt development of the DA sensory neurons of different classes, in particular ddaA, ddaB, ddaD, ddaE and ddaF neurons. This was not surprising given the strong defects detected in development of the entire dendritic arborization neuron cluster in response to mutated *CSN5* (Figure 1C, D).

**Figure 3:**
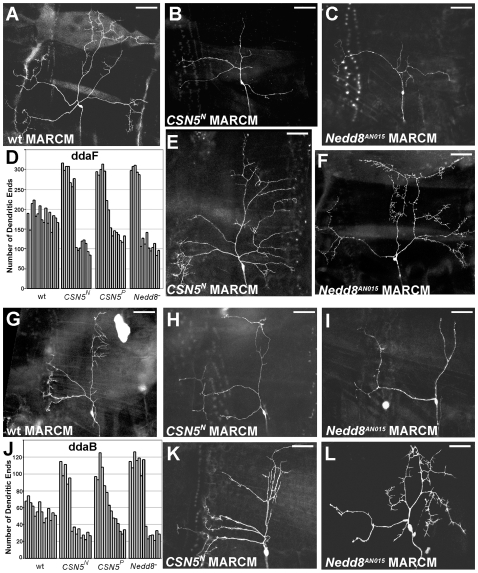
Loss of CSN function affects different classes of the sensory neurons. (A, G) Wild type ddaF and ddaB neurons, respectively. Mutations in *CSN5* inhibit dendritic branching in ddaF (B) and ddaB (H) neurons. Similarly, the *Nedd8*-mutant ddaF (E) and ddaB (K) neurons are frequently less elaborated. Over-branching phenotype in ddaF and ddaB neurons caused by loss of *CSN5* (E, K) or *Nedd8* (F, L). Scale bar: 50 µm. Quantifications of terminal dendritic ends in wild type, *CSN5^N^* and *Nedd8* mutant ddaF (D) and ddaB (J) neurons.

The MARCM clones of either *CSN5* or *Nedd8* in these classes of neurons demonstrated similar phenotypic groups in dendritic branching: either reduction or stimulation of dendritic branching (Figure 3). We calculated the numbers of dendritic ends and detected characteristic distribution of the increased and decreased branching phenotypes (Figure 3). The fact that mutations in *CSN5* and *Nedd8*, the invariable components of the CSN machinery, result in identical defects indicates that CSN is involved in common mechanisms of control of neuronal development.

### Cullin1 and Cullin3 have opposite effect on dendritic branching

The CSN machinery undoubtedly participates in multiple processes in cellular regulation. The CSN5 and Nedd8 proteins are members of the common and substrate-nonspecific part of the CSN system. Mutated proteins would lead to broad and pleiotropic effects. In turn, mutated Cullins would generate more exclusive phenotypes, representing particular parts of the broad *CSN5* or *Nedd8* mutant phenotypes. Therefore, to dissect the complex effect of CSN in dendrites, we tested the downstream components of CSN – the Cullin proteins.

First we investigated whether *CSN5* and members of the *Drosophila* Cullin family interact genetically in the DA neurons. In our previous experiments, the hypomorphic *CSN5* mutations provided sensitive genetic background and demonstrated strong genetic interactions with several genes [18], [20]. We took similar approach here to examine genetic interactions between CSN5 and Cullin components of the CSN-mediated machinery. We generated double heterozygous for *CSN5* and different *cullins* to investigate whether reducing doses of *CSN5* and individual Cullins generate specific “narrow” phenotypes in dendritic development.

**Figure 4:**
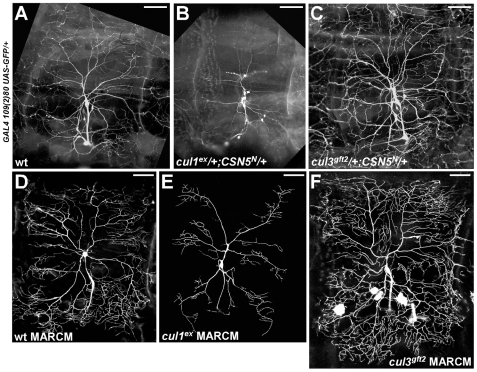
CSN acts in neurons via Cullin1 or Cullin3. (A) PNS neurons in a non-mutant third instar larva, visualized by the *109(2)80-GAL4*-driven expression of *UAS-GFP*. (B) *CSN5* and *cullin1* interact genetically, simultaneous reduction of their functions represses dendritic development. (C) *CSN5* and *cullin3* interact genetically, *CSN5/cul3* double heterozygotes show increased dendritic elaboration. Single *CSN5, cullin1* or *cul3* heterozygous have no effect on dendritic development (not shown) (D) MARCM clone of a wild type ddaC neuron. (E) Loss of *cullin1* lead to decreased branching. (F) *cullin3*-mutant ddaC neurons demonstrate abnormal over-branching phenotype. Scale bar: 50 µm.

We identified that *CSN5* and *cullin1* genetically interact in dendritic development. Double heterozygotes for *CSN5* and *cullin1* demonstrate a simple, “narrow”, phenotype of inhibited dendritic arborization (Figure 4B; 4A - control). Single *CSN5* or *cullin1* heterozygotes did not affect dendritic development (data not shown). Apparently, simultaneous reduction of the CSN and Cullin1 functions led to the impaired downregulation of the CSN^Cullin1^ targets that control the branching pattern. Thus, normally CSN5 and Cullin1 appear to act in concert to promote dendritic branching.

**Figure 5:**
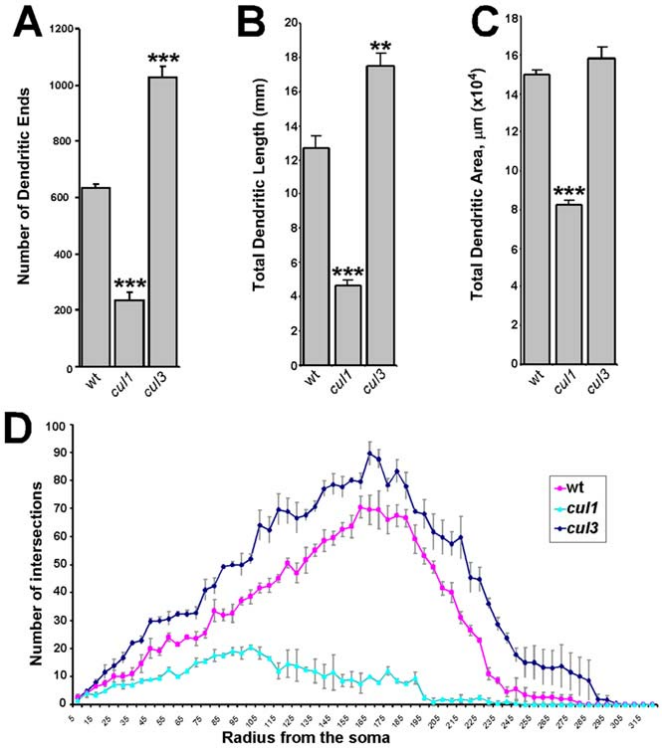
Cullin1 and Cullin3 have opposite effects on dendritic arborization. (A) Average number of the terminal dendritic ends in wild type, *cul1* and *cul3* mutant ddaC neurons. (B) Quantifications of total dendritic length in wild type, *cul1* and *cul3* mutant ddaC neurons. (C) Quantifications of total dendritic area in wild type, *cul1* and *cul3* mutant ddaC neurons. (D) Sholl analysis histogram of dendritic arbors of wt, *cul1* and *cul3* ddaC clones. Error bars represent standard deviation.

To confirm that Cullin1 is involved in dendritic branching, we generated MARCM clones of *cullin1*. As expected, we found that loss of *cullin1* caused a reduction of dendritic elaboration and fewer terminal dendritic ends (Figure 4E, 4D – control; Figure 5A). Loss of *cullin1* was overlapping only with the first part of the dual *CSN5*-mutant phenotype, suggesting that CSN stimulates dendritic branching via Cullin1.

We also identified the Cullin component that acts oppositely to Cullin1 in dendritic branching. In particular, we detected that double heterozygotes for *CSN5* and *cullin3* show some increase of dendritic arborization (Figure 4C). Single *CSN5* or *cullin3* heterozygotes did not alter branching. This *CSN5/cullin3* genetic interaction suggests that CSN normally prevents dendrites from over-branching via Cullin3-mediated proteolysis.

To prove this hypothesis, we generated MARCM clones for two strong independent alleles of *cul3*. We observed that loss of *cul3* led to significant dendritic over-branching (Figure 4F, Figure 5A). These findings suggest that CSN represses dendritic branching via control of Cullin3 function. We describe the CSN/Cullin3 pathway in neuronal development in details in a separate study (59).

To further investigate the effect of *cul1* and *cul3* mutations on dendritic branching, we employed several quantificational approaches. We calculated total dendritic length of ddaC neurons in wild type, *cul1* and *cul3* mutants. As expected, total dendritic length was severely decreased in *cul1* mutants and increased in *cul3*-mutant ddaC neurons, when compared to wild type (Figure 5B). Next we examined the total dendritic area by fitting a polygon around the most distant end points of ddaC neurons and then measuring the area. We detected that dendritic area was strongly reduced in *cul1* mutants (Figure 5C). When compared to wild type, *cul1*-mutant ddaC neurons covered only 59.3% of the area (p<0.001). The changes in *cul3* clones dendritic area were less dramatic. We detected that an average *cul3*-mutant ddaC neuron covers 106.8% of the average wild type ddaC neuron area, but the difference was not statically significant (p = 0.11) (Figure 5C).

Dendrite morphology was further analyzed by the Sholl analysis [44] that measures the complexity of dendrites as a function of distance from the soma, or the cell body (Figure 5D). Briefly, a series of concentric circles were centered on the cell body at 5 µm intervals, and the number of dendrites intersecting each circle was counted. In control and *cul3* mutant ddaC neurons the number of branches was increasing progressively from proximal to distal reaching the peak between 160–185 µm from the cell body. However, the number of intersections was higher in *cul3* mutants at any distance from the cell body. The maximum number of branches intersecting Sholl circle was also significantly higher in *cul3* mutants (89.7±3.5) than in control (70.5±3.9). In contrast, *cul1*-mutant neurons exhibited dramatic reduction in the number of intersections at all distances (maximum 20.5±1.4) without the obvious peak. In addition, in *cul1* mutants, branches were only detected at a shorter distance from the cell body (up to 245 µm) compared to control (up to 280 µm), while in *cul3* mutants dendritic branches were detected at the slightly higher distance from the cell body (up to 300 µm). This correlated with the observed differences in dendritic areas measured by the polygon method.

Next we used Reversed Strahler analysis [45] to analyze branching pattern and complexity of the ddaC neurons. We detected that a typical wild type ddaC neuron contains 6 branch orders (Table 1). In *cul1* mutants, dendritic arbors were generally simpler. Specifically, all observed *cul1*-mutant ddaC neurons had no more than 5 branch orders. We also found that loss of *cul1* predominantly affected the higher order branches (Table 1). In contrast, dendrites in *cul3-*mutant clones were more complex, if compared to wild type (Table 1). The *cul3* neurons also exhibited additional, new branch order. In particular, we frequently detected 7, vs 6 in wild type, branch orders in *cul3*-mutant ddaC neurons. Besides, loss of *cul3* led to proportional increasing of the branch number in each order (Table 1).

**Table 1:** Reversed Strahler analysis of the ddaC neurons.

genotype	1st order	2nd order	3rd order	4th order	5th order	6th order	7th order (terminal)
wt (3)	-	1	4.3±0.7	14±2.6	44.5±5.7	159.5±13.1	652.5±48.7
*cul1^ex^*(5)	-	-	1	3.7±0.7	13.7±2.1	57±7.8	252±70.2
*cul3^gft2^*(3)	0.7±0.6	2	7±1	21.7±1.6	77.7±8	298.7±26.6	1198±139.1

Values are the mean (± standard deviation) number of total dendritic branches in each order. The total number of neurons observed is indicated in parentheses. “–” indicates order that was not observed for the particular genotype.

In summary, these results revealed that *cul3* mutants exhibit proportional dendritic overgrowth. Dendritic tree in *cul3* mutants is more complex with significantly increased branch density. In contrast, *cul1* mutants demonstrate simpler dendritic trees, shrinking dendritic arbor, decreased branch density and preferential inhibition of the higher order branching. Taken together, our data suggest that Cullin3 normally acts to prevent dendritic overgrowth, while *cul1* acts to promote dendritic branching.

### Loss of the F-box protein Slimb represses dendritic branching

To narrow down the downstream targets of the CSN/Nedd8/Cullin1 pathway, we investigated whether the substrate-specific components of the Cullin1-based ubiquitin ligases, the F-box proteins, are involved in dendritic morphogenesis. In contrast to constitutive Nedd8 and Cullin1 components of the SCF ubiquitin ligases, the F-box proteins are variable and provide specificity to the ligase complex.

**Figure 6:**
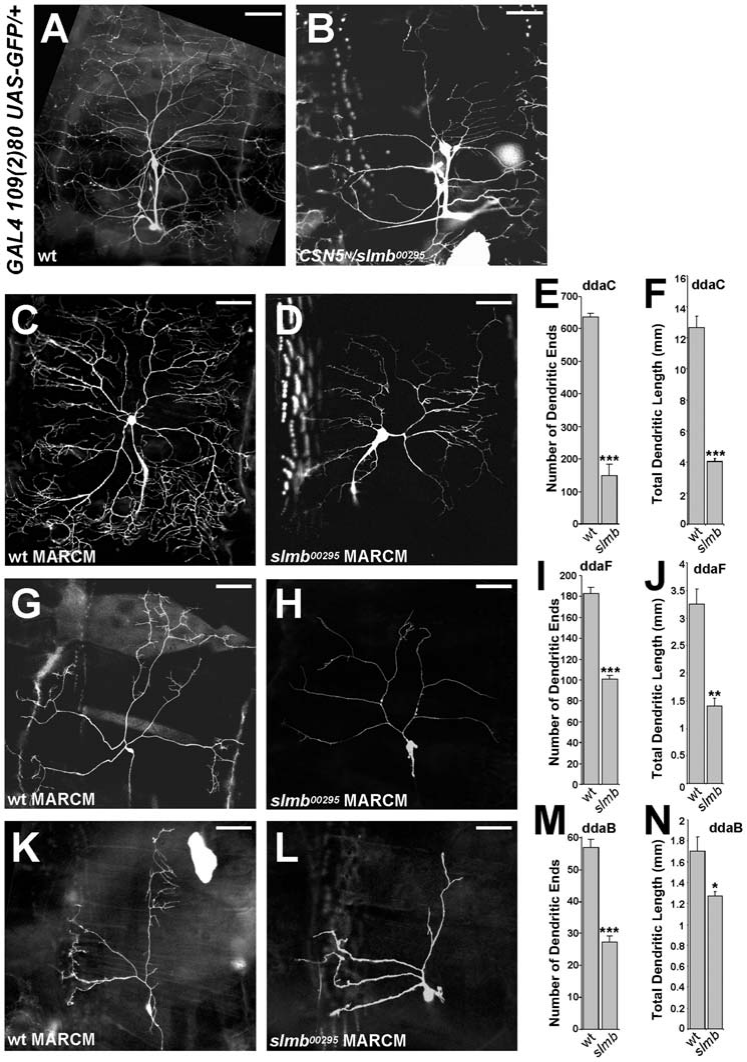
The F-box protein Slimb is involved in dendritic branching. (A) PNS neurons in a non-mutant third instar larva, visualized by the *109(2)80-GAL4*-driven expression of *UAS-GFP*. (B) *CSN5* and *slmb*, a component of the SCF complex, interact genetically in dendritic development leading to reduced dendritic branching. (C, F, I) Typical wild type ddaC, ddaF, ddaB neurons, respectively. (D, G, J) *slmb*-mutant mosaic clones of ddaC, ddaF, ddaB neurons with the characteristic fewer branching phenotype. Scale bar: 50 µm. (E, H, K) Quantifications of terminal dendritic ends in wild type and *slmb* mutant ddaC, ddaF and ddaB neurons. ***: p<0.001, **: p<0.01, *: p<0.03.

We tested several known F-box proteins for dominant genetic interactions with *CSN5* in dendrites. We found that simultaneous removal of single copies of the F-box protein Slimb (Slmb) and CSN5 had an inhibiting effect in dendritic development (Figure 6B; 6A – wild type). Slmb has been reported to be involved in Cullin1-dependent proteolysis in several systems [46], [47], [48], however function of Slmb in the nervous system has not been previously identified. We found that MARCM clones for mutants in *slmb* gene led to severe reduction of dendritic branching (Figure 6D, H, L) vs control (Figure 6C, G, K), suggesting that function of the *Drosophila* F-box protein Slmb is critical for dendrites. Detailed quantificational analysis of ddaB, ddaC, ddaF neurons confirmed that loss of *slmb* causes reduced dendritic branching (Figure 6E, I, M). Total dendritic length was also reduced in *slmb* mutant clones (Figure 6F, J, N). Since we only observed strong repression in dendrite development in *slmb* mutants, we concluded that *slmb* shares the reduced branching phenotype seen in *CSN5*, *Nedd8* and *cullin1*.

### Cubitus interruptus, a substrate for SCF^Slmb^, is involved in neuronal development

The F-box proteins of the Cullin1-based ubiquitin ligases directly interact with the substrate proteins to target them for destruction. To further investigate how CSN/SCF^Slmb^ is involved in dendritic morphogenesis, we looked for downstream targets of Slmb. Loss of *slmb* function would lead to accumulation of these nondegraded target proteins. Therefore, we tested the subcellular levels of previously reported targets of Slimb in *slmb*-mutant MARCM clones. In addition, in an attempt to mimic the *slmb* phenotype, we overexpressed the known target proteins in PNS neurons.

Several proteins have been reported to be targeted for degradation by Slmb; among them is beta-catenin/Armadillo (Arm). Arm could be one of the most suitable candidates because it has already been reported to participate in dendritic morphogenesis [49]. However, we could not detect significant changes in dendrite morphology when wild type or activated forms of Arm were overexpressed in larval PNS.

**Figure 7:**
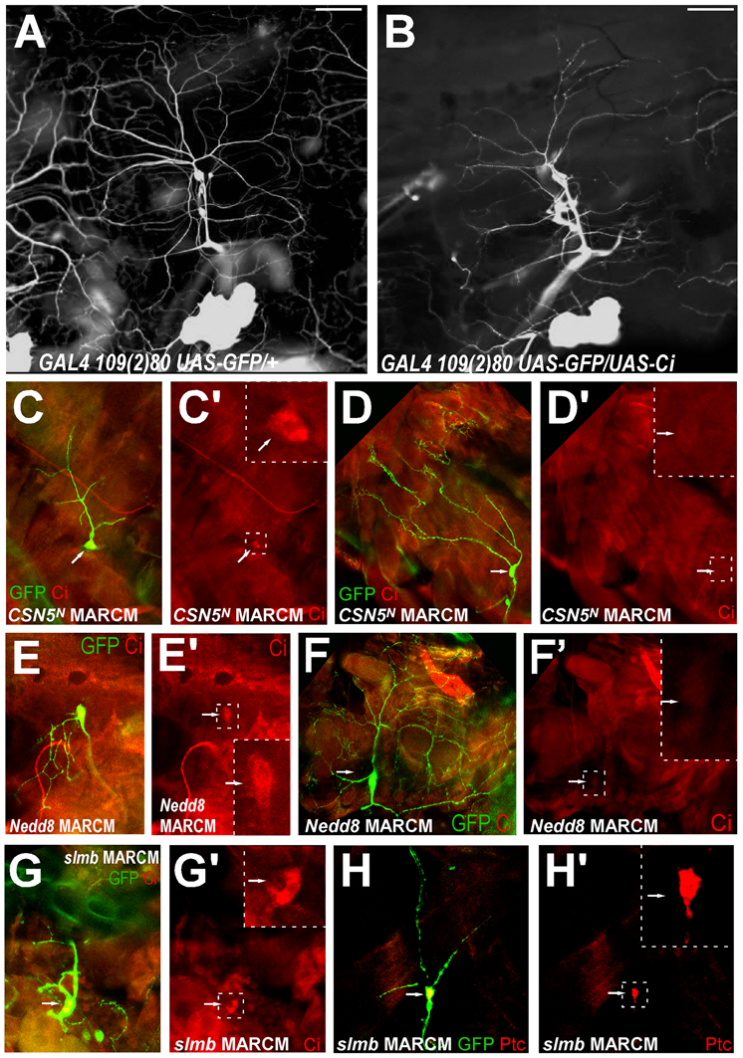
Overaccumulation of Ci inhibits dendritic development. PNS neurons in a non-mutant third instar larva, visualized by the *109(2)80-GAL4*-driven expression of *UAS-GFP*. (B) *109(2)80-GAL4*-driven overexpression of Ci, one of the Cullin1^Slimb^ targets, leads to reduced dendritic branching. (C, E) Ci is over-accumulated in *CSN5*- or *Nedd8*-mutant neurons with severely repressed dendritic branching. (D, F) Levels of Ci are lower in *CSN5*- or *Nedd8*-mutant neurons without the strong reduction of elaboration. (G) Ci is over-accumulated in *slmb* MARCM clones. (H) Levels of Patched, one of the Hh targets, are elevated in *slmb*-mutant neurons. Scale bar: 50 µm.

Unexpectedly, we found that expression of the upstream activation sequence (UAS) construct of full-length Cubitus interruptus (Ci), one of the Slimb targets, caused a strong reduction in dendrite elaboration and a shrinking dendritic field, suggesting that Ci is a target for SCF^Slmb^-dependent degradation in PNS neurons (Figure 7B; 7A – control).

Immunostaining with antibodies to Ci further supported the overexpression data. The Ci protein was frequently accumulated in *CSN5* or *Nedd8* MARCM clones (Figure 7C, E), while it was virtually undetectable in adjacent non-mutant neurons. Apparently, normal concentration of Ci in neurons is low due to the strict control of CSN/Cullin1^Slmb^-dependent degradation. Failure to downregulate Ci leads to the inappropriate elevation levels of Ci, which evidently acts to prevent neurons from extending branches.

We also observed that Ci accumulation correlated with the degree of dendritic branching defects. Ci levels were always higher in cell bodies of the *CSN5*- or *Nedd8*-mutant neurons with fewer branches (Figure 7C, E), when compared to the relatively lower levels in the *CSN5*- or *Nedd8*-mutant neurons with elaborated branching (Figure 7D, F). We counted the clones with arbitrary strong or weak accumulation of Ci. In *CSN5*-mutant clones, we found 17 and 11 examples for each group, respectively. In *Nedd8* clones, we detected 9 cases of strong, and 7 cases of weak Ci accumulation. In a few cases, clones with different phenotypes and distinct levels of Ci were detected in the same larvae. These arbitrary groups showed distinct dendritic branching patterns: the degree of branching suppression was higher in clones with strong Ci accumulation, and lower in clones where Ci levels were weaker. We never detected strong accumulation of Ci in neurons with nearly normal or increased dendritic elaboration.

In addition, we immunostained the *slmb* clones with anti-Ci to strengthen the connection between the strong under-branching phenotype and accumulation of Ci. As expected, we detected that Ci was always over-accumulated in *slmb* clones (Figure 7G).

Cubitus interruptus is the zinc finger transcription factor that has been known as the primary target of Hedgehog (Hh) signal transduction [50], [51]. Relevantly here, *ci* was found in the RNAi screen to identify transcription factors required for proper morphogenesis of *Drosophila* sensory neuron dendrites [52]. However, the functional role of Ci in dendrites has not been elucidated. Our findings indicate that Ci is involved in maintaining the proper balance of dendritic wiring under the control of the CSN-controlled proteolysis.

Previous analysis has shown that *ci* is required for the activation of Hh targets such as *patched* (*ptc*) [53]; and that ectopic expression of *ci* stimulated *ptc* expression [54], [55], [56]. To examine whether the levels of Ptc are altered in *slmb*-mutant clones, we stained them with anti-Ptc. We detected that Patched was over-accumulated in the *slmb*-mutant neurons (Figure 7H). This result suggests that in *Drosophila* PNS Ci acts, at least in part, as a component of the Hh pathway.

## Discussion

Here we report a previously unrecognized role of the COP9 signalosome in regulating dendritic morphogenesis. We found that altering CSN function in *Drosophila* larval neurons leads to a highly complex phenotype. In particular, mutations in *CSN5* or *Nedd8*, the key invariable components of the CSN machinery, caused either inhibition or stimulation of dendritic elaboration. In contrast, we found that mutations in the variable CSN system components generated uncomplicated “narrow” phenotypes. In this report, we showed that mutations in Cullin1/Slmb^Ci^ repressed dendritic branching. In a separate study, we described that loss of another Cullin-based ligase, Cullin3^Kelch^, stimulated additional dendritic branching (59). These findings demonstrate for the first time that the CSN system has a high profile role in dendritic development. Given the previously detected connection between CSN and human neurological disorders, our model study is the first step in understanding of its mechanism.

### CSN is a major player in the intrinsic developmental program

Investigating how neurons define their morphology is crucial for understanding the anatomical cause of genetic disorders or the reasons neurons fail to regenerate after injury or disease. Despite intensive studies, our knowledge about the intrinsic regulatory program that directs dendritic morphogenesis is incomplete. Undoubtedly, intrinsic dendritogenesis is under the multistep control of transcription factors, actin cytoskeleton remodeling molecules, micromolecule synthesis and turnover, etc. Our study has begun to uncover the integrative regulatory network in control of these intracellular factors.

Our finding that null mutations in *CSN5* or *Nedd8* caused two very distinct phenotypes of either under- or over-branching might suggest the existence of a certain threshold or switch in the intrinsic dendritic developmental program. We hypothesize that such threshold would divide CSN-mediated dendritic development hierarchically into earlier and later phases. To pass the threshold and enter the later phase, a neuron should successfully accomplish the earlier developmental phase. It appears that in our study the earlier phase is based on Cullin1-mediated events, because their failure caused the under-branched part of the phenotype. This explains the predominantly *cullin1*-mutant phenotype in *CSN5* and *Nedd8* strong mutants, despite the fact that Cullin3 function was also affected in these mutants. Indeed, loss of CSN function affects all Cullin-based programs but phenotypically results in the hierarchically earlier phase. This interpretation suggests a simple and economic mechanism of CSN-mediated control so that passing to the next phase in development would simply require a substitution of one Cullin to another while keeping the regulatory CSN machinery intact.

There could be alternative or additional mechanisms of the Cullin1/Cullin3 interaction during dendritic morphogenesis. For example, there is a possibility of a differential requirement for the two cullins at different distances from the cell body. However, this explanation is not supported by the Sholl analysis. In particular, in *cul1* and *cul3* mutants we did not detect disproportional changes in branch distribution (Figure 5D), suggesting that *cul1* and *cul3* act independently of distance from the cell body. Alternatively, it is possible that Cullin1 and Cullin3 operate simultaneously, acting to oppose the effect of each other. If this model were true, we would detect an intermediate phenotype in strong *CSN5* and *Nedd8* mutants. However, this phenotype was not observed in strong *CSN5* or *Nedd8* mutants (Figure 2H, 3D, 3J).

The two distinct phenotypes detected in *CSN5* or *Nedd8* strong mutants can be explained by the technology of generating mosaic clones. After genetic recombination, there is always some amount of the wild type proteins left in the cells of the mosaic clones. The perdurance of the pre-recombinant wild type CSN5 or Nedd8 proteins in post-recombinant mosaic clones for *CSN5* or *Nedd8* mutations in some cases could allow neurons to successfully execute the Cullin1-mediated earlier program and pass the presumable threshold. Then they would initiate the next, Cullin3-dependent later developmental phase. However, by the time the cells initiate this phase, the cytoplasmic CSN5 or Nedd8 proteins could be already used up, preventing the successful execution of the Cullin3-dependent program. This would result in extensive dendritic over-branching, reproducing the *cul3*-mutant phenotype. In contrast, in hypomorphic *CSN5* mutants, expression of the CSN5 protein would often allow to complete the Cullin3 phase, leading to an “intermediate” branching phenotype. This hypothesis is supported by the finding that perdurance of the CSN5 and Nedd8 proteins is increased at 18°C thereby weakening the resulting mutant phenotype [18], [20].

On the other hand, the temperature dependence effect might also be a reason for another explanations, not related to perdurance. In particular, different susceptibility of the CSN/Cullin1^Ci^ and CSN/Cullin3^Kelch^ complexes to lower temperature might be responsible for the more frequent over-branching phenotype detected at 18°C. Alternatively, there could be uneven requirements for the CSN levels between different cullins, Cullin1 and Cullin3 in particular.

### CSN and neurological disorders

Members of the CSN machinery have been detected in multiple neurological syndromes. In particular, altered components of the COP9 signalosome have been detected in Smith-Magenis syndrome [29], [31], Down syndrome [33], Alzheimer's disease and Parkinson's disease [34], Machado-Joseph neurodegenerative disease [36], [37], and X-linked mental retardation syndrome [38], [39]. The detection in our study of Cubitus interruptus in dendritic morphogenesis is intriguing, because Ci has also been reported to be involved in neurological disease. Truncation mutations of the *GLI3* zinc finger transcription factor, a human homologue to Cubitus interruptus, can cause Greig cephalopolysyndactyly syndrome, a pleiotropic, multiple congenital anomaly syndrome with low frequency central nervous system anomalies, hernias, and cognitive impairment [57].

Given our finding of the central role of CSN in dendritic morphogenesis and the high evolutional conservatism of the CSN pathway, it is tempting to speculate that these different human disorders have a common CSN-dependent mechanism. Based on the results of our model study, we expect that malfunctions in CSN function would lead to pleiotropic effects in dendritic density in the human brain wiring and contribute to different neuropathological changes.

It has to be noted that dendritic abnormalities associated with many forms of mental disorders and neurodegenerative diseases may be the only evident pathology in some of these disorders. However, dendritic defects are likely to be accompanied by multiple defects at the molecular level, in signaling pathways, metabolism, etc. In case of impaired CSN function these defects are expected, given the involvement of CSN and protein degradation in virtually every major aspect of cellular regulation. Therefore, if mutated, CSN might have strong, broad effects on brain function. A combination of altered dendritic density with defects in other cellular mechanisms underlying the entire profile of determinants of neuronal function would synergistically contribute to disease.

## Materials and Methods

### Fly Stocks

Canton S and *w^1118^* were used as standard strains. The following fly stocks were used: *CSN5^L4032^* (or *CSN5^P^*, a hypomorphic allele resulting from a P-element insertion); *CSN5^N^* (null allele); *CSN5^quo1^*; *Nedd8^AN015^*; *Nedd8^AN024^*; *cul1^ex^* (*lin19^ex^*); *cul3^gft2^* (*gft^2^*); *cul3^06430^* (*gft^06430^*), *slimb^00295^*, *UAS-mCD::GFP*; *C155*-*GAL4*, *UAS-mCD::GFP, hsFLP*; *tubP-Gal80*, *FRT40A*/*CyO, 109(2)80-GAL4, UAS-GFP*. *Nedd8^AN015^*; *Nedd8^AN024^*; *cul1^ex^* were kindly provided by Dr. Cheng-Ting Chien. Other stocks were obtained from the Bloomington *Drosophila* Stock Center.

### Single Neuron Analysis

To generate single cell CSN5 or slmb mutant clones flies *C155-GAL4*, *UAS-mCD::GFP, hsFLP*; *tubP-Gal80*, *P{neoFRT}82B* were crossed with *C155-GAL4*, *UAS-mCD::GFP, hsFLP*; CSN5* (or slmb*) P{neoFRT}82B/TM3B, Sb. The *Nedd8*- and *cullin1*-mutant clones were generated in a similar way using 40AFRT and G13FRT chromosomes, respectively.

For MARCM clones, embryos from these crosses were collected at 25°C or 18°C. To induce mitotic recombination, embryos were heat-shocked in 37.5°C water bath for 40 minutes. The embryos were incubated at 25°C or 18°C and third instar larvae were examined for GFP-labeled clones. Only clones in abdominal segments 3–5 were imaged and quantified to ensure consistency.

### Immunohistochemistry

For immunostaining, third instar larvae containinig single neuron clones were collected, dissected, fixed in 3.7% formaldehyde for 15 minutes and permeabilized in 0.1% Triton X-100 in phosphate-buffered saline. Monoclonal antibodies to Ci were kindly provided by Dr. Robert Holmgren. anti-Patched was obtained from the Developmental Studies Hybridoma Bank.

### Quantitative morphological analysis

The number of dendritic ends was counted manually. Total dendritic area was defined as a polygon between most distal dendritic ends and measured in the ImageJ software (NIH). NeuronStudio [58] was used to measure total dendritic length and analyze neuron branch order. Dendrites were traced semi-automatically with careful manual corrections. The Strahler method was used as described previously [45]. Sholl Analysis Plugin for ImageJ (Ghosh Lab, UCSD) was used for Sholl analysis. A series of concentric circles were centered on the soma at 5 µm intervals, and the number of dendrites intersecting each circle was calculated. For some measurements neurons were traced manually. For *cul1*, *cul3* and *slmb* mutant clones, data are presented as means±SD. All statistical analyses for *cul1*, *cul3* and *slmb* mutant were performed using Student's t-test.
